# Application of Infrared Thermography in Early Screening of Sepsis and Prediction of Septic Shock Risk: A Systematic Review

**DOI:** 10.1155/emmi/1104018

**Published:** 2026-02-06

**Authors:** Wei-Ya Du, Jun-Ting Huang, Zuo-Peng Zhang

**Affiliations:** ^1^ Department of Emergency Medicine, The First Affiliated Hospital of Guangzhou Medical University, Guangzhou, 510000, China, gzhmc.edu.cn; ^2^ Department of Critical Care Medicine, The Second Affiliated Hospital of Shantou University Medical College, Shantou, 515000, China, st120.cn

**Keywords:** infrared thermography, sepsis, septic shock, shock, thermal imaging

## Abstract

**Background:**

Infrared thermography (IRT), a noninvasive imaging modality capable of capturing micron‐level temperature variations (resolution up to 0.03°C) and generating thermal maps, has demonstrated unique value in dynamically reflecting physiological and pathological states. Recent studies have explored its potential as a diagnostic tool for sepsis. This review assesses the feasibility of IRT in detecting sepsis and septic shock.

**Methods:**

A systematic literature search was conducted across PubMed, Web of Science, and Scopus databases using combinations of keywords (“infrared thermography,” “thermal imaging,” “sepsis,” “septic shock,” “shock”) in titles, abstracts, or topics. Articles published after 2015 were included.

**Results:**

After applying exclusion criteria, 11 studies were analyzed. Most findings highlighted IRT’s efficacy in early sepsis detection, disease progression monitoring, and prognostic evaluation.

**Conclusion:**

IRT serves as a valuable tool for early diagnosis and monitoring of sepsis, with significant potential for broader clinical adoption. Further standardization and technical refinement are required to enhance its reliability in critical care settings.

## 1. Introduction

Sepsis, a life‐threatening organ dysfunction caused by a dysregulated host response to infection [1], remains a critical global health challenge with high morbidity and mortality. Globally, an estimated 49 million sepsis cases and 11 million sepsis‐related deaths were reported in 2017, accounting for 19.7% of total mortality [2]. While advancements in fluid resuscitation and antimicrobial therapy have improved outcomes, mortality rates remain alarmingly high at 29.7% [2]. Early detection and intervention are paramount for improving prognosis, yet conventional diagnostic methods relying on clinical signs, laboratory markers, and imaging studies face limitations, including invasiveness, time delays, and reliance on complex infrastructure [3]. Failure in early detection and timely intervention may precipitate progression to septic shock, multiorgan failure, and mortality [4]. While numerous studies have investigated emerging strategies for sepsis detection—including biomarker‐driven approaches such as sepsis index [5] and microRNA profiling [6]—the inherent clinical complexity and patient heterogeneity continue to challenge the accuracy and generalizability of current early recognition and severity assessment methodologies.

Over the past decade, infrared thermography (IRT) has emerged as a noninvasive imaging modality capable of detecting microvascular perfusion abnormalities through micron‐level temperature resolution (up to 0.03°C) and dynamic thermal mapping. Infrared thermal imaging (IRTI) technology operates on the physical principles of infrared radiation emitted from object surfaces. By capturing infrared radiation within the 8–14 μm wavelength emitted by the human body and employing computational image processing, it generates intuitive pseudocolor thermal maps to visualize surface temperature distribution [7]. As homeothermic mammals, humans maintain core temperature stability through sophisticated thermoregulatory mechanisms involving neural circuits, vascular networks, and eccrine glands, with minimal environmental thermal influence via perspiration and respiratory heat exchange [8]. Pathophysiological alterations manifest as localized temperature deviations between diseased and healthy tissues, where symmetrical and stable thermal patterns characterize physiological states, while metabolic abnormalities or circulatory disruptions induce measurable thermal field anomalies [9]. The foundational premise lies in correlating regional temperature variations with hemodynamic, metabolic, and neurophysiological activities—such as localized temperature elevations in inflammatory [10] or neoplastic [11] regions due to hyperperfusion, contrasted with hypothermic signatures in ischemic or thrombotic zones [9].

IRTI offers noninvasive, real‐time, and dynamic monitoring capabilities, permitting precise detection of subtle thermal fluctuations to evaluate microcirculatory dynamics and metabolic states [12]. Recent advancements have expanded its clinical utility across diverse medical domains: early screening for breast cancer [13] and cutaneous malignancies [11], diagnostic assessments in musculoskeletal [14], neurological [15], and vascular disorders [9], and quantitative evaluation of burn injuries [16], trauma, inflammatory conditions [17], and nociceptive responses [18]. The growing adoption of IRTI highlights its emerging role in sepsis screening and prognostication. However, no systematic review currently synthesizes existing evidence on IRTI’s diagnostic and prognostic value in sepsis management, underscoring the necessity for comprehensive investigation.

## 2. Literature Review

### 2.1. Search Strategy

This systematic review has been registered on the International Prospective Register of Systematic Reviews (PROSPERO) and strictly adheres to the Preferred Reporting Items for Systematic Reviews and Meta‐Analyses (PRISMA) guidelines [19], with the PRISMA checklist provided in the table below (Table1). A comprehensive literature search was conducted across PubMed, Web of Science (WoS), and Scopus using combinations of the following MeSH terms and keywords: infrared thermography, thermal imaging, sepsis, septic shock, and shock. Additional manual searches were performed through reference lists of relevant articles.

**Table 1:** PRISMA checklist.

Section	Item	Checklist item description	Location in manuscript
TITLE	1	Identify the report as a systematic review	Title, Abstract
ABSTRACT	2	Structured summary of background, objectives, methods, results, and conclusion	Abstract
INTRODUCTION	3	Describe the rationale for the review	Section 1
INTRODUCTION	4	Provide an explicit statement of objectives	Section 1
METHODS	5	Eligibility criteria (inclusion/exclusion)	Section 2.2
METHODS	6	Information sources (databases, dates)	Sections 2.1 and 2.2
METHODS	7	Search strategy (keywords, Boolean)	Table 2
METHODS	8	Selection process (screening, inclusion)	Section 3.1, Figure 1
METHODS	9	Data collection process	Section 2.3
METHODS	10	Data items collected	Sections 3.3 and 3.4
METHODS	11	Risk of bias in individual studies	Section 3.2, Table 4
METHODS	12	Effect measures (qualitative and quantitative)	Sections 3.3 and 3.4
METHODS	13	Synthesis methods (qualitative thematic synthesis using CFIR)	Sections 3.3, 3.4, Table 3
RESULTS	14	Study selection and flow diagram	Section 3.1, Figure 1
RESULTS	15	Characteristics of included studies	Table 3
RESULTS	16	Risk of bias in studies	Section 3.2
RESULTS	17	Results of individual studies	Sections 3.3, 3.4, Table 3
RESULTS	18	Synthesis of results (barriers, facilitators, models)	Section 3.4
DISCUSSION	19	Summary of evidence	Section 4
DISCUSSION	20	Limitations of the evidence	Section 4
DISCUSSION	21	Conclusions and implications	Section 5
OTHER	22	Registration Status	Section 2.1
OTHER	23	Sources of funding and support	Acknowledgment
OTHER	24	Competing interests	Declaration

### 2.2. Inclusion and Exclusion Criteria

#### 2.2.1. Inclusion Criteria

a. Human studies
b. Published between February 2015 and February 2025 in English
c. Original research articles (prospective/retrospective cohorts, clinical trials, and case reports)
d. Focus on IRTI applications to sepsis or septic shock using trunk/limb imaging

#### 2.2.2. Exclusion Criteria

a. Non‐English publications, systematic reviews, meta‐analyses, books, conference abstracts, and nonhuman/animal studies
b. Studies without IRTI‐based sepsis detection via trunk/limbs

### 2.3. Data Extraction

Data extraction focused on study design, IRTI parameters (device specifications and measurement sites), clinical endpoints (early detection, monitoring, and prognosis), and statistical outcomes. Two independent reviewers resolved discrepancies through consensus.

### 2.4. Quality Assessment

The risk of bias was assessed using the Joanna Briggs Institute (JBI) critical appraisal checklists.

## 3. Results

### 3.1. Study Selection

**Figure 1:**
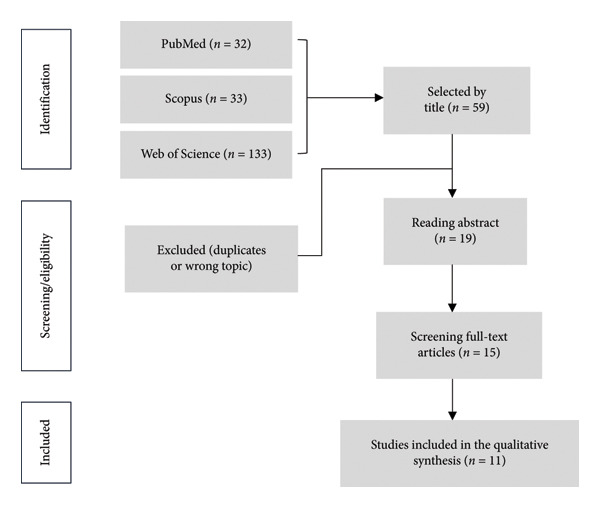
PRISMA flow diagram.

A comprehensive search across PubMed, WoS, and Scopus identified 198 candidate studies (Table 2), of which 139 studies were excluded due to title discrepancies or duplication with the included literature. The abstracts of 59 documents were then read and analyzed, and only 19 documents made it to the full‐text readings, of which 4 were excluded because they did not match the subject matter. Of this literature, only 11 were ultimately included in this systematic review (Figure 1: PRISMA flow diagram). All selected studies were clinical trials investigating IRTI applications in sepsis and septic shock detection (Table 3).

**Table 2:** Database search strategy.

Database	Search strategies	Data	Results
PubMed	(“sepsis”[MeSH Terms] OR “pyemia”[Title/Abstract] OR “pyosepticemia”[Title/Abstract] OR “septicopyemia”[Title/Abstract] OR “blood poisoning”[Title/Abstract] OR “bloodstream infection”[Title/Abstract] OR “septic shock”[Title/Abstract] OR “shock”[Title/Abstract]) AND (“infrared thermography”[Title/Abstract] OR “infrared image”[Title/Abstract] OR “thermal imaging”[Title/Abstract] OR “infrared sensor technology”[Title/Abstract])	March 2025	32

Scopus	((TITLE‐ABS‐KEY (“infrared thermography”) OR TITLE‐ABS‐KEY (“infrared image”) OR TITLE‐ABS‐KEY (“thermal imaging”) OR TITLE‐ABS‐KEY (“infrared sensor technology”))) AND ((TITLE‐ABS‐KEY (sepsis) OR TITLE‐ABS‐KEY (pyemia) OR TITLE‐ABS‐KEY (pyosepticemia) OR TITLE‐ABS‐KEY (septicopyemia) OR TITLE‐ABS‐KEY (“septic shock”) OR TITLE‐ABS‐KEY (shock) OR TITLE‐ABS‐KEY (“blood poisoning”) OR TITLE‐ABS‐KEY (“bloodstream infection”))	March 2025	33

WoS	(“infrared thermography” (Topic) or “infrared image” (Topic) or “thermal imaging” (Topic) or “infrared sensor technology” (Topic)) AND (sepsis (Topic) or pyemia (Topic) or pyosepticemia (Topic) or septicopyemia (Topic) or “septic shock” (Topic) or shock (Topic) or “blood poisoning” (Topic) or “bloodstream infection” (Topic))	March 2025	133

**Table 3:** Key studies on IRTI applications in sepsis and septic shock.

Authors (year)	IRTI application	Measurement site	Study description	Key results & conclusions
Coats et al. (2018)	Fever vs. sepsis differentiation	Upper limb	IRTI comparisons of upper arm, forearm, hand, and finger regions across 56 subjects (controls, febrile, and sepsis groups).	Significant arm‐to‐finger gradient in controls (Δ2.75°C, *p* < 0.0001), absent in sepsis/fever groups. Temperature differences outperformed absolute values for differentiation.
Sethi et al. (2019)	Hemodynamic shock prediction	Abdomen–thigh	Analyzed 253 thermal images from pediatric ICU patients using IRTI‐derived core‐peripheral gradients.	Machine learning models achieved 75% (0 h), 77% (3 h), and 69% (12 h) accuracy in shock prediction, demonstrating noncontact feasibility.
Hasanain et al. (2019)	Early sepsis recognition	Ear–nose–core	Facial thermal asymmetry (ETD) measured in 11 ICU patients and 4 controls during cold stress testing.	ETD declined progressively 36 h before sepsis onset (vs. stable in controls), suggesting utility for ICU monitoring.
Vanshika et al. (2022)	Shock risk stratification	Whole body	Deep learning analysis of 103,936 thermal video frames (406 clips from 22 PICU patients).	LSTM models predicted shock 5 h prior to onset using center‐peripheral gradients (CPD) and heart rate integration.
Jing et al. (2022)	Hypoperfusion severity assessment	Legs	Calculated thermal heterogeneity parameters (SD, entropy, low‐temperature area ratio) in 373 high‐risk patients.	Thermal metrics matched capillary refill time (CRT) in prognostic accuracy (AUC = 0.88), supporting noninvasive severity staging.
Sigita et al. (2023)	Mortality risk evaluation	Knee–upper thigh	IRTI patterns correlated with mottling scores in 81 septic shock patients within 24 h of ICU admission.	Anterior thigh/patellar IRTI patterns showed prognostic potential when adjusted for mottling (OR = 4.1).
Amson et al. (2020)	Day‐8 mortality prediction	Multiple sites	Core‐to‐skin gradients assessed in 61 septic shock patients during the initial 24 h.	Gradient > 7°C independently predicted Day‐8 mortality (aHR = 3.2, 95% CI 1.4–7.3, *p* < 0.05).
Hasanin et al. (2024)	Postoperative mortality exclusion	Toe–canthal	Serial IRTI gradients measured in 56 surgical septic shock patients at 0, 6, and 12 h.	Toe temperature ≤ 25.5°C at 12 h provided 70% NPV for mortality, though inferior to serum lactate (PPV = 92%).
Luo et al. (2023)	AI‐enhanced risk modeling	Legs	Deep learning (ResNet) analysis of leg IRTI in 373 high‐risk patients (including sepsis cohort).	Model achieved 84% sensitivity, 91% specificity for mortality prediction, pending sepsis‐specific validation.
Gutowski et al. (2024)	COVID‐19 sepsis prognostic value	Finger	Compared IRTI fingertip metrics vs. CRT/renal perfusion in 102 COVID‐19 sepsis patients.	CRT and renal perfusion independently predicted outcomes (*p* < 0.001); fingertip IRTI lacked prognostic significance.
Ferraris et al. (2018)	IRTI‐mottling score correlation	Knee	Analyzed IRTI temperatures vs mottling scores in 46 septic shock patients pre/post resuscitation.	Mottled knees showed lower temperatures (*p* = 0.02), but neither parameter predicted 28 days of mortality.

### 3.2. Risk of Bias

The results of the JBI quality assessment and bias risk analysis of 11 observational studies related to thermal imaging demonstrated that all included studies carried a low risk of bias with overall good research quality, and the assessment pass rate ranged from 90.9%–100% (Table 4). Specifically, 9 studies (Nagori et al. [20]; Vats et al. [21]; Luo et al. [22]; Kazune et al. [12]; Amson et al. [23]; Hasanin et al. [24]; Luo et al. [25]; Gutowski et al. [26]; Coats et al. [27]) performed excellently across all 11 assessment dimensions—including baseline similarity between groups, consistency in exposure measurement, rationality of group allocation, reliability of outcome indicators, completeness of follow‐up, and ethical compliance—without obvious methodological flaws. In contrast, only 2 studies (Al‐Sadr et al. [28]; Ferraris et al. [29]) had one item rated as “No,” attributable to insufficient identification and control of potential confounding factors (e.g., age and underlying diseases); however, this minor limitation did not compromise the overall research quality or alter the low bias risk classification. Collectively, these findings indicate that existing observational studies on thermal imaging adhere to high‐quality research standards in methodological design, data collection, statistical analysis, and ethical compliance, thereby providing reliable support for evidence synthesis in relevant systematic reviews.

**Table 4:** Risk of bias analysis by JBI tool for observational studies.

Author	Q1	Q2	Q3	Q4	Q5	Q6	Q7	Q8	Q9	Q10	Q11	%	Risk
Nagori et al. (2019)	Y	Y	Y	Y	Y	Y	Y	Y	Y	Y	Y	100	Low
Al‐Sadr et al. (2019)	Y	Y	Y	N	Y	Y	Y	Y	Y	Y	Y	90.9	Low
Vats et al. (2022)	Y	Y	Y	Y	Y	Y	Y	Y	Y	Y	Y	100	Low
Luo et al. (2022)	Y	Y	Y	Y	Y	Y	Y	Y	Y	Y	Y	100	Low
Kazune et al. (2023)	Y	Y	Y	Y	Y	Y	Y	Y	Y	Y	Y	100	Low
Amson et al. (2020)	Y	Y	Y	Y	Y	Y	Y	Y	Y	Y	Y	100	Low
Hasanin et al. (2024)	Y	Y	Y	Y	Y	Y	Y	Y	Y	Y	Y	100	Low
Luo et al. (2023)	Y	Y	Y	Y	Y	Y	Y	Y	Y	Y	Y	100	Low
Gutowski et al. (2024)	Y	Y	Y	Y	Y	Y	Y	Y	Y	Y	Y	100	Low
Ferraris et al. (2018)	Y	Y	Y	N	Y	Y	Y	Y	Y	Y	Y	90.9	Low
Coats et al. (2018)	Y	Y	Y	Y	Y	Y	Y	Y	Y	Y	Y	100	Low

*Note:* Q1: Similarity of groups, Q2: Exposure measured similarly, Q3: Group allocation, Q4: Confounding factors, Q5: Reliability of outcomes, Q6: Follow‐up complete, Q7: Outcome assessment, Q8: Follow‐up losses, Q9: Statistical analysis, Q10: Exposure time, Q11: Ethical considerations, Yes: score 1, No: score 0, TOTAL Score 11, Low risk: score > 70%, Moderate risk: score 50%–70%, High risk: < 50%.

### 3.3. Application of IRTI in Early Detection of Sepsis and Septic Shock

Five studies investigated IRTI’s utility for early sepsis detection, primarily focusing on temperature gradient analysis to identify microcirculatory dysfunction. Coats et al. [27] analyzed upper limb thermal gradients in 56 patients (control, febrile, and sepsis groups). Healthy controls exhibited a marked temperature drop from upper arm to fingertips (Δ2.75°C), whereas febrile (*p* = 0.944) and septic (*p* = 0.710) groups demonstrated gradient loss, suggesting peripheral‐core thermal equilibration. The study emphasized that limb temperature gradients—rather than absolute values—hold discriminatory potential for sepsis detection in emergency settings. Nagori et al. [20] employed machine learning to analyze abdominal‐to‐thigh temperature gradients in pediatric ICU patients, achieving 75%–77% accuracy in predicting hemodynamic shock within 3 h. Al‐Sadr et al. [28] introduced an IRTI‐based sepsis recognition protocol leveraging core‐to‐peripheral temperature disparities. Through cold immersion experiments simulating sepsis‐induced thermoregulatory responses, facial imaging of inner canthus–nasal tip differentials demonstrated the capacity to detect pathognomonic thermal patterns 36 h pre‐onset in ICU patients, establishing its utility for presymptomatic identification. Vats et al. [21] implemented long short‐term memory (LSTM) models to analyze pediatric ICU thermal videos, integrating central‐to‐peripheral temperature difference (CPD) with heart rate variability for 6‐h septic shock predictions. The framework yielded an AUROC of 0.81 ± 0.06 and precision–recall AUC of 0.78 ± 0.05 at 5 h preshock intervals, enabling clinically actionable early interventions. Luo et al. [25] assessed perfusion severity in 373 critically ill patients using leg thermal heterogeneity parameters: standard deviation (SD), skewness, kurtosis, entropy, and low‐temperature area ratio (LTAR). SD and LTAR matched capillary refill time (CRT) in prognostic accuracy for mortality prediction (AUROC = 0.866 when combined). Critically, these parameters correlated with CRT (*r* = 0.73), lactate (*r* = 0.68), and blood pressure trends, positioning IRTI as a noninvasive alternative for shock severity stratification.

### 3.4. IRTI for Prognostic Evaluation in Sepsis and Septic Shock

Six studies evaluated IRTI’s prognostic capacity for mortality prediction and disease progression:

Kazune et al. [12] examined skin temperature patterns in 81 septic shock patients within 24 h of ICU admission. While knee and anterior thigh temperatures alone showed no survival correlation (*p* ≥ 0.05), their combination with mottling scores revealed significantly lower overall temperatures in nonsurvivors (*p* = 0.02). Adjusted models identified anterior thigh thermal patterns as independent predictors of 28‐day mortality (OR = 4.1, 95% CI: 1.2–8.9), supporting IRTI’s adjunctive role in postresuscitation monitoring. Amson et al. [23] investigated core‐to‐skin temperature gradients in 61 septic shock patients, measuring differences between core body temperature and peripheral sites (fingers, toes, knees, and forearms). A gradient exceeding 7°C at the index finger independently predicted 8‐day mortality after SOFA score adjustment. This gradient demonstrated correlations with established hypoperfusion markers, such as CRT, mottling scores, and arterial lactate levels, supporting IRTI as an objective noncontact method for peripheral perfusion evaluation and short‐term mortality prediction. Hasanin et al. [24] assessed central‐to‐peripheral temperature gradients in 56 postoperative septic shock patients. While toe temperature ≤ 25.5°C at 12 h showed 70% negative predictive value for in‐hospital mortality, its predictive capacity was inferior to serum lactate and CRT (both exceeding 90% positive predictive value). Luo et al. [25] employed deep learning algorithms to analyze thermal heterogeneity parameters (e.g., LTAR, SD) from leg IRTI images in 373 high‐risk critically ill patients. The ResNet model achieved optimal performance in mortality risk prediction, demonstrating IRTI’s capability for noninvasive, high‐accuracy prognostic assessment. Contrasting findings emerged from Ferraris et al. [29], who analyzed thigh/knee temperatures in 46 septic shock patients. Despite observing significantly lower knee temperatures in patients with mottling, neither mottling scores nor IRTI‐derived temperatures predicted 28‐day ICU mortality. Similarly, Gutowski et al. [26] found no prognostic value for finger IRTI in 102 COVID‐19‐associated sepsis patients, where CRT and renal cortical perfusion (RCP) emerged as independent predictors of deterioration and mortality (*p* < 0.001).

## 4. Discussion

IRTI is emerging as a promising noncontact, noninvasive, safe, rapid, and dynamic diagnostic imaging modality [7]. Its application in sepsis and septic shock detection is expanding extensively. However, systematic reviews evaluating IRTI’s role in sepsis and septic shock detection remain absent. This review aims to investigate current research progress and explore the latest advancements of IRTI technology in this field.

The majority of studies included in this review validate IRTI’s robust performance in early screening, disease monitoring, and prognostic evaluation of sepsis and septic shock. Compared to alternative diagnostic methods, portable IRTI devices exhibit distinct advantages. First, IRTI is particularly suitable for prehospital settings and low‐resource regions where advanced imaging modalities like ultrasound, CT, and MRI are often unavailable. Second, IRTI features simple and rapid operation without requiring extensive specialized training. Finally, IRTI is compatible with low‐cost thermal cameras, enabling applications in diverse healthcare settings, including hospitals, community clinics, nursing homes, and even home care [30].

The physiological mechanisms underlying IRTI‐based sepsis detection remain incompletely elucidated. Sepsis is pathophysiologically characterized by systemic inflammatory responses and microcirculatory dysfunction triggered by infections [1]. Inflammatory foci typically exhibit localized temperature elevations detectable by thermal imaging for rapid localization and dynamic monitoring (Figure 2). The progression involves pathogen invasion activating immune responses, massive cytokine release (“cytokine storm”), vascular dilation, endothelial injury, and microthrombosis, leading to tissue ischemia–hypoxia. Persistent hypoperfusion and hypoxia disrupt cellular metabolism, causing lactate accumulation, acidosis, and ultimately multiorgan failure [31]. Septic shock is defined as sepsis requiring vasopressors to maintain MAP ≥ 65 mmHg despite fluid resuscitation, accompanied by blood lactate ≥ 2 mmol/L [1]. Studies reveal that even after achieving hemodynamic stabilization, tissue hypoperfusion often persists in septic shock patients, driving organ dysfunction development [32]. Emerging evidence demonstrates detectable microcirculatory and peripheral circulatory impairment during early septic shock [33], with the severity and duration of microcirculatory dysfunction critically influencing prognosis [34, 35]. Reduced peripheral perfusion serves as an early marker of microcirculatory compromise in septic shock [36, 37], making its assessment imperative. Current peripheral perfusion evaluation [38] methods include skin temperature, CRT, mottling score, peripheral perfusion index, serum lactate, lactate clearance rate, central venous oxygen saturation, central venous–arterial CO2 gap, laser Doppler flowmetry [39], near‐infrared spectroscopy [40], and handheld orthogonal polarization spectral imaging [41].

Figure 2Infrared thermal imaging of sepsis and localized inflammation. (a) Characteristic sepsis pattern: Diminished core‐to‐periphery thermal gradient (Δ*T* < 1.0°C). (b) Focal inflammatory response: Localized hyperthermia (Δ*T* + 2.5°C) in the nasopharyngeal region.

**Figure:**
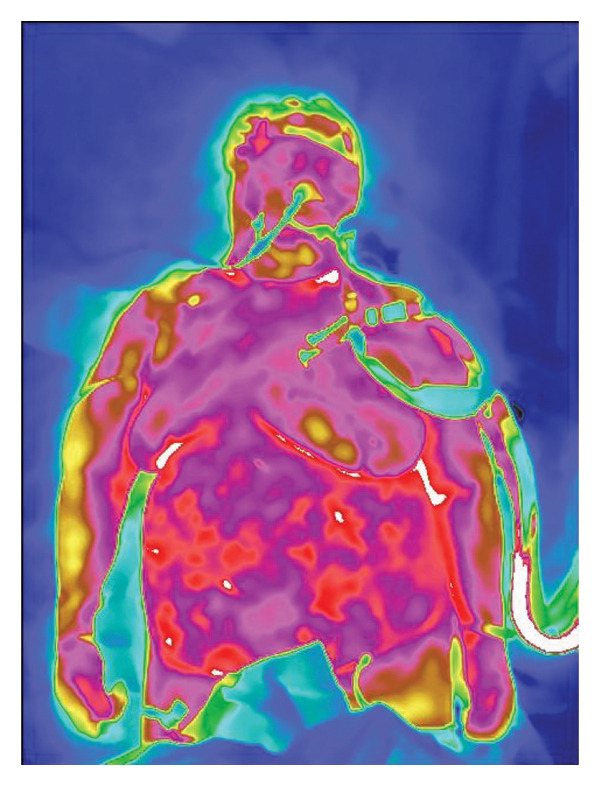
(a)

**Figure:**
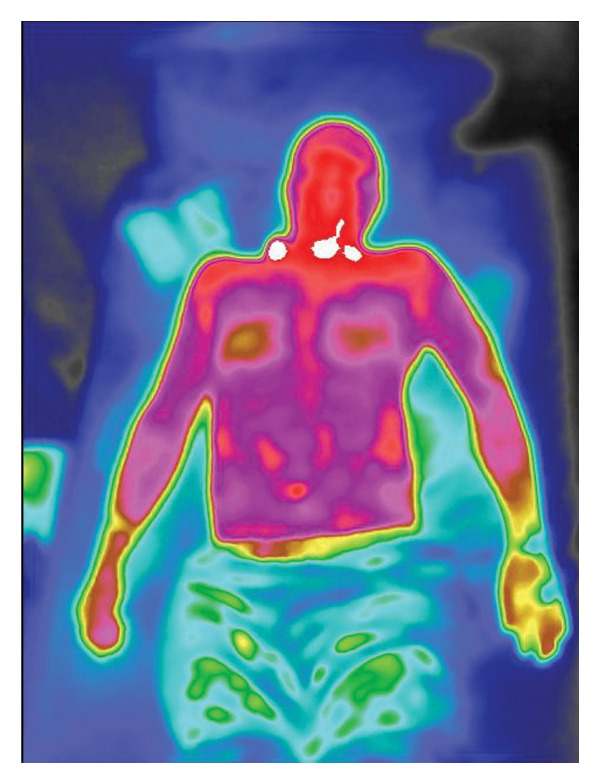
(b)

Peripheral skin temperature provides a reliable indicator for tissue perfusion assessment. Cooling extremities and large skin temperature gradients correlate with severe organ failure and poor survival in sepsis [23, 42]. Porcine model studies by Pereira et al. demonstrate significant associations between skin temperature distribution parameters (SD and spatial gradients) and shock indices/mean arterial pressure, validating IRTI’s capability to monitor acute circulatory impairment [43]. Bourcier et al. identified toe‐to‐ambient temperature gradients as predictive biomarkers of prognosis in septic shock [42]. Leante‐Castellanos et al. established persistent CPDs (> 2°C for ≥ 4 h) as sensitive early diagnostic markers for late‐onset neonatal sepsis [44]. Magnin et al. further confirmed IRTI’s utility in detecting early peripheral perfusion changes through porcine septic models [45].

For early detection, most studies in this review utilized IRTI to analyze central‐to‐peripheral temperature gradients. Coats et al. [27] identified altered upper limb temperature gradients distinguishing febrile and septic patients. Al‐Sadr et al. [28] validated core‐to‐limb temperature differentials as sepsis predictors through cold pressor tests. Nagori et al. [20] achieved early septic shock detection via CPD, while Vats et al. [21] demonstrated predictive capabilities using thermal maps in pediatric ICUs. Luo et al. [22] reported comparable prognostic accuracy between unilateral leg thermal heterogeneity parameters and conventional CRT measures.

Regarding prognostic evaluation, most studies focused on single‐site thermal imaging (thigh, knee, and finger) or core‐to‐peripheral gradients. Amson et al. [23] identified core‐to‐fingertip temperature gradients > 7°C as independent mortality predictors on Day 8. Conversely, Ferraris et al. [29] and Kazune et al. [12] found no mortality correlation despite lower knee temperatures in mottled patients. Luo et al. [25] developed a deep learning–based mortality prediction model using thermal heterogeneity analysis. Hasanin et al. [24] reported moderate predictive value for 12‐h toe temperatures, though inferior to serum lactate and CRT. Notably, Gutowski et al. [26] found no significant prognostic value in fingertip IRTI for COVID‐19‐associated sepsis.

Despite encouraging results, several limitations warrant attention. First, small sample sizes due to sepsis/septic shock case scarcity necessitate future multicenter trials. Second, labor‐intensive data processing highlights the need for automated analytical models to enhance clinical translation. Finally, integrating IRTI with emerging diagnostic tools and developing novel algorithms could further improve diagnostic accuracy.

This review acknowledges methodological limitations: (1) inclusion of all available studies regardless of design quality due to limited IRTI‐sepsis research; (2) only five studies directly comparing IRTI with conventional diagnostics, precluding conclusive superiority claims.

In conclusion, IRTI demonstrates significant potential for early detection, monitoring, and prognostic evaluation of sepsis/septic shock, offering prospects for sepsis prevention and mortality reduction through timely intervention.

## 5. Conclusion

IRTI emerges as a viable adjunctive tool for sepsis management, offering noninvasive, real‐time insights into microcirculatory dysfunction and peripheral perfusion dynamics. Current evidence underscores its efficacy in early detection, severity stratification, and mortality risk prediction. Nevertheless, widespread clinical adoption necessitates resolving environmental interference, protocol standardization, and algorithmic validation in heterogeneous populations. With technological refinements and expanded multicenter studies, IRTI holds promise as a scalable solution for improving sepsis outcomes worldwide.

## Ethics Statement

Ethical approval and consent to participate were not required for this study, as it is a systematic review of previously published data.

## Consent

This article does not contain any personal information that could lead to the identification of the patient(s); therefore, consent for publication was not required.

## Disclosure

All authors reviewed and approved the final version.

## Conflicts of Interest

The authors declare no conflicts of interest.

## Author Contributions

Wei‐Ya Du conceptualized and drafted the original manuscript; Jun‐Ting Huang conducted critical revisions and language editing; Zuo‐Peng Zhang (corresponding author) provided scientific oversight and finalized the manuscript structure.

## Funding

This work was supported by the Guangdong Province Undergraduate Innovation and Entrepreneurship Training Program Project (No. 202A022), titled “Infrared Thermography Distribution Characteristics and Expression Analysis in Severe Sepsis with Pneumonia.”

## Data Availability

Data sharing is not applicable to this article as no datasets were generated or analyzed during the current study.
